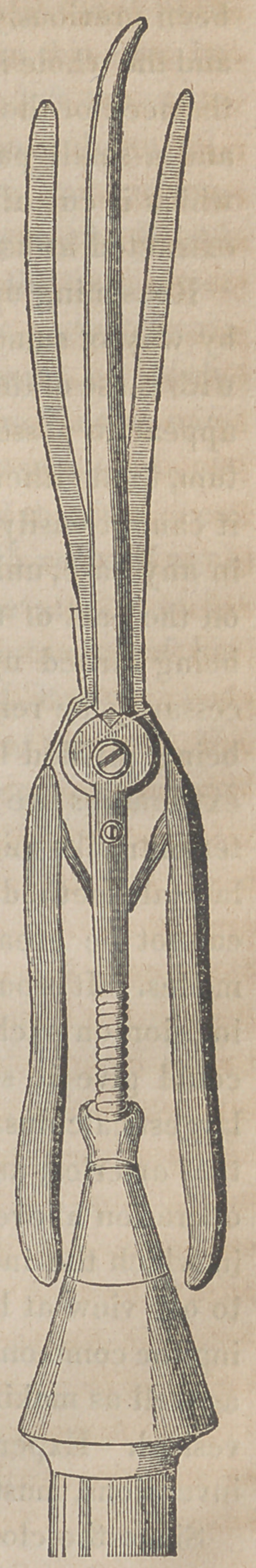# Operations for Calculi

**Published:** 1834

**Authors:** A. Hermange


### Art. IV.—Operations for the Removal of Urinary Calculi.

*Some observations on the various means of relief that have been proposed, or adopted in cases of Urinary Calculi. Read before the Cincinnati Medical Society, by* A. Hermange, M. D.

Of all the physical ailments, incident to our nature, no one has more occupied the zealous and indefatigable attention of the profession than stone in the bladder. The circumstances attending their presence, particularly in the male bladder, are indeed of so painful, and distressing, and formidable a character to the patient himself, and when considered in reference to remedial operations, are so trying and perplexing to the professional attendant, that it is not astonishing that medical science and art, always devoted to the cause of human suffering, should be exhausted in devising ways and means of relief.

Every method must, from the nature of the case, answer one or the other of two great indications,—either to destroy the calculus, or lessen its irritation, whilst in the bladder;—or to cut a passage for its extraction. We speak, of course, of all cases, where the stone, from its dimensions, cannot escape through the natural, or dilated passage of the urethra.

Accordingly, in medical history, we find various lithontriptics recommended to answer the first, or more merciful indication, which, it was hoped, might dissolve urinary calculi; or at least check or stop their further increase;—various menstrua which, by being injected into the bladder, might effecttheir decomposition and solution, or mere solution. But it has unfortunately been found by experience, that although concentrated chemical agents in a short time, or largely diluted ones in a longer period, can effect those objects in the chemical laboratory; still, when similar agents are made to exert their influence through the general circulation, they will no doubt be modified, in their progress to the bladder, by the chemical influence of the fluids of the system itself; and the only good we can hope for, (and that even is problematical,) is to arrest their further progress, by altering the chemical character of the animal fluids. In a concentrated form, the remedy would in a short time, prove itself worse than the disease. We are therefore compelled to use such articles (whether introduced into the stomach, or injected into the bladder) in a highly diluted state, and then, simply for the purpose of palliating the symptoms, and lessening irritation. The health and vigor of the constitution would give way, infallibly, under the continued treatment necessary to make much impression upon the stone. We learn from the writings of Dr. Whytt, that the minister Walpole was cured by the remedy of Mrs. Stephens; but his death, at a later period, was attributed to the effects produced on his system, by the too great quantity of soapy, or alkaline matter required for the destruction of the calculus. Dr. Hartley too, the great advocate of Mrs. Stephens' nostrum, is said to have died of the disease, after ineffectually taking more than two hundred pounds of soap, the main ingredient in her medicine. All that we can now say of this case, is, that it is wonderful, how his constitution held out so long under such treatment, and that supposing his system to have been affected with a phosphatic diathesis, we know not how far the exhibition of acescent lithontriptics, to an equal extent, would have availed.

At that period the public attention was all alive to the important subject: but little, however, was done. Subsequently, as chemical science was developed, Scheele, by the discovery of the uric acid in the most common varieties of stone, directed the attention of other distinguished men to the subject. And the luminous researches of Wallaston, Fourcroy, Vauquelin, Pearson, Brande, Marcet, Prout, Henry, Thenard, &c., certainly inform us of the true chemical nature of these urinary concretions; but they likewise teach us caution, with regard to the proper methods of correcting calculous diathesis. In the urine, according to Mr. Brande, uric acid and the earthy phosphates are always present, and these last are mainly held in solution by their excess of acid. If then, as Mr. Brande has suggested, a concretion of uric acid were present in the bladder, and we were to administer some alkaline solution, with the view of forming a soluble urate, the exhibition of it, beyond this point, might likewise neutralize the excess of phosphoric acid, and give rise to a precipitation of the earthy phosphates, which might go to form an additional crust around the calculus, before this last could be reached by the dissolving alkali. On the other hand the exhibition of acids, for the purpose of arresting any further precipitation of the earthy phosphates, and of removing those already formed, has, after some time, occasioned a deposition of uric acid. We might indeed, by the exhibition of alkaline remedies, carried to a certain extent, lessen the irritation produced by the mulberry calculus, chiefly composed of the oxalate of ammonia, by favoring a precipitation on its rough, unequal surface, of a smooth layer of phosphate of ammonia and magnesia; but this is only correcting one evil, by the substitution of another, in the increase of volume, which must render the ultimate aid of surgery more precarious.

Chemistry then indeed has enlightened the practitioner; but it has only done so by exposing, in a clearer point of view, the difficulties of the subject. It teaches us, (as has been already remarked,) that nothing can be hoped for, in the way of dissolving large calculi at least; and only urges prudence in our attempts to correct depraved habits of body. This is, however, an important point gained; for although chemistry has left such sufferers to nearly all the horrors of their situation, still, they are not subject to have those ills aggravated by zealous and humanely intended; but, at the same time, blind and ignorant interference.

Having a full view of the insufficiency of acid, or alkaline menstrua to dissolve calculi, as proposed by Fourcroy, Vauquelin and others, and of their tendency, when persevered in, to provoke inflammation, as well as, when unscientifically administered, of sometimes enlarging the stone; M. Jules Cloquet, with a praiseworthy enthusiasm, but guided more by feelings of humanity, than by a firm conviction of its eventual utility, offered to substitute pure, or distilled water, either as a menstruum, or to act by attrition. For this purpose, he had an apparatus constructed at the hospital of St. Louis in Paris, which failed, to be sure, in accomplishing the desired object; but which has been, for some years back, employed advantageously in other affections of the bladder. It consisted of a double sound introduced into the urethra, one of the tubes of which was connected, by the intervention of a long conduit of gum elastic, with a reservoir somewhat elevated:—by this means a constant supply of water, at a temperature little rising of 100 degrees of Fahrenheit, was kept up to the bladder. A similar conduit, connected with the other tube of the sound, carried off the water, after it had served its purpose. No doubt can be entertained, that a current of water, kept up a sufficiently long time, would at length wear away a calculus even of the hardest texture, as even rocks, in the beds of rivers, show its slow powers of attrition, and even of solution:—but however bland and unirritating the fluid might be, and how long soever the bladder itself might bear such an affusion, no one, in this way, could in sober earnest, expect success. We have mentioned, however, the plan of M. Cloquet, for the double purpose, of referring to the apparatus at the hospital of St. Louis, as a standing monument of the inefficacy of all menstrua to dissolve voluminous calculi, at least whilst lying loose in the bladder, and of adverting to the benefit to be derived by similar affusion, to a moderate degree, in certain chronic irritations and catarrhal affections of that viscus.

As in all cases of vital importance to mankind, the human mind is, *at all times*, active in unfolding the resources of nature, it very often happens, that the discoveries of the passing moment belong really to times past; and that kindred intellects, of different periods, by striking upon the same vein of thought, arrive at the same conclusions; without any knowledge on the part of the last investigator of what was done before. In this way, frequently, the learned lore of bygone ages, which from fortuitous circumstances, had either been lost, or buried under the accumulated mass of intellectual rubbish, is reclaimed; and although not, in this case, with the *intended* beneficial result to suffering humanity, still, in many cases, (and we will notice some in the sequel of these observations,) bringing honor and fame to (we will call them) the discoverers themselves, and momentous advantage to mankind. A mode of treatment, precisely similar to that of Jules Cloquet, was published in London in the year 1740 by Stephen Hales; and a few years afterwards the work was translated into French; but of this J. Cloquet certainly knew nothing. The plan has had its first sleep, and as a remedy for calculous affections, may now sleep forever.

The first meditations of Dr. Civiale, in the years 1817 and 1818, on the subject of saving calculous patients from the surgical knife, resulted in devising a plan, by which he hoped to be able to dissolve calculi, enclosed in a bag, introduced into the bladder. A straight sound was first to be introduced into the urethra, and through this, another tube, with a double conduit, was to be passed. The inner extremity of this latter tube was armed with two branches, to which the open end of the bag was attached. In this way he could, at pleasure, open and shut the bag, somewhat after the manner of the clasp of a purse. One of the conduits of the inner tube communicated with the bag, and through this the lithontriptic liquid was to be passed; the other communicated with the interior of the bladder, by which the urine could be voided; or if occasion required it, some liquid could be thrown into that viscus, capable of neutralizing the action of the lithontriptic, in case any of it should escape from the bag internally. But it must be evident, that the almost positive certainty of this occurring, (independently of the permeable character of the bag itself,) constituted the principal vice of his first method. In order to determine the nature of the solvent to be used, he proposed to employ another instrument previously, by which drilling out portions of the calculus, he could submit the detritus to chemical analysis. This accesary, and purely mechanical method of destroying stone, soon, however, took up his whole attention, as the only practicable one in his estimation, and the attempt at solution was abandoned.

A few words will, in the subsequent part of these observations, be devoted to the "Procede Civiale," which, although not adequate to all cases, has proved, and will always prove extensively useful to the unfortunate portion of mankind concerned, has sheda brilliant lustre upon French surgery, and gained imperishable fame for its author. For obvious reasons, the original idea of Civiale failed, and drew upon it and all similar attempts, in the report to the Royal Academy of Sciences, the character of "une espece de chimere." It was given up to pursue another, that promised immediate success, and without the least exertion to correct, if possible, radical defects—defects in the mechanical conception, and in the preparation of materials or making the bags.

Whilst the writer of these observations was in Paris, a modification of Civiale's original plan was, however, proposed by M. Robinet, which had at least the merit of considerable ingenuity, whatever may be the final judgement upon it by the profession. It was condemned by Dr. Civiale himself, at that time, as having come to no practical result; but this was at least, a premature judgement, as M. Robinet was then only making his investigations, and had merely given to the world the rudiments of his plan.*

It was a contrivance, by which a *long* bag open at one end, and closed at the other, and made of some suitable material, is conveyed into the bladder, through a straight sound, previously introduced into the urethra. In the open end the stone

*The ensuing account of M. Robinet's plan has been principally taken from the Repertoire Generale d'Anatomie, &c.

is caught, and as the same end is withdrawn externally, it must of necessity slip to the closed extremity, in which it will be retained in the inside of the bladder. By a method analogous to that of M. Cloquet, referred to above, a current of acid, or alkaline, or any other liquor, of a proper strength, can be brought to act upon it, without coming into contact with the surface of the bladder, under any circumstances, except through the texture of the bag itself.

Now it must be confessed, at first sight, that M. Robinet's plan is likewise beset with difficulties, the least of which, perhaps, is the mechanical adaptation of the instruments. But let it be put down to the eternal honor of the profession, that suffering humanity is never left to its fate, until all the stores of nature and art are expended in vain for its relief; and that in the series of remedial measures, harsh and dangerous processes are only adopted, when, after the most patient and laborious research, nothing safer, or less painful can be devised. The modest, and candid, and disinterested manner in which M. Robinet proposed his method, certainly merits much praise: and although it may not be found adequate to the purpose in all cases, or in any case, still it deserves the attentive consideration of the profession. It was offered with a thorough knowledge of all the difficulties surrounding it; but with an enthusiastic determination to overcome them himself, if possible, and with an earnest recommendation, that others competent to the task should likewise take up the subject.

1
2
3
4
5
6
7
10
8
9
11

The mechanical part of his plan may be stated, in a few words, in reference to the figures.

Fig. 1. Is a cylindrical, membranous bag, prepared from the cœcum of a sheep; naturally closed at one of its extremities. Its length should be about one foot. Its diameter varible, according to circumstances.

Fig. 2. A cylindric tube, to one end of which, is attached a highly elastic, circular spring, to which the open end of the bag is to be fixed. The circular spring must of course have a larger diameter than that of the calculus.

Fig. 3. Represents the bag fixed to the spring.

Fig. 4. Stilet, one part of which, possessing considerable elasticity, is so made that, when left to itself, it will bend round, like the spiral spring of a watch. The spiral portion of it should be at least ten inches long.

Fig. 5. Straight sound, one foot long, which can allow of the introduction through it of the stilet.

Fig. 6. Canula to be introduced into the urethra and which will permit the ingress and egress of the rest of the apparatus. It should have a diameter of about three lines and a half, and nine inches in length.

Fig. 7. Shows the elliptic form which the circular spring (Fig. 2. to which the open end of the bag is attached) assumes, in passing through the canula, Fig. 6.

Fig. 8. Represents the closed end of the bag, pushed by the end of the spiral spring of the stilet, and both escaping to the interior of the bladder. As they enter, the spiral spring, being left free, will wind itself up, together with the bag.

Fig. 9. Represents the bag and spiral spring, escaped entirely into the bladder. The open end of the bag is likewise seen distended by the circular spring of Fig. 2.

Fig. 10. Disposition of the apparatus, when the calculus has been introduced into the bag, and the open end of this drawn out externally.

Fig. 11. Represents the apparatus in action. A sound, with a double conduit, is seen to permit the free ingress and egress of the dissolving menstruum into that portion of the bag containing the stone. A thermometer regulates the temperature of the lithontriptic solvent.

The different parts of the apparatus are to be used thus;—After moistening the membranous bag, and oiling it, as well as all the other instruments, the stilet (Fig. 4.) is to be passed into the straight sound, (Fig. 5.) by straightening out the spiral portion of it. Both are then to be passed into the bag, in such a manner, that the end of the sound and stilet will touch the closed extremity of the bag. The whole is then to be pushed into the canula, (Fig. 6.) which must be previously introduced into the urethra. When the extremity of the bag has reached the interior of the bladder, the straight sound, which has hitherto controlled the elasticity of the stilet, is to be withdrawn. The elasticity of the circular spring, (of Figs. 2 and 3.) is now to be so far overcome, that it will enter the canula, and by pushing it to the interior of the bladder, at the same time that the stilet is advanced inwards, the whole of the bag will be made to enter. As the bag enters it will be wound upon the spiral spring; and when the whole of it has been introduced, its open end will be dilated by the circular spring, (see Fig. 9.) The operator will then endeavor to catch the stone in the bag, and when this is accomplished, the circular spring and stilet are to be withdrawn together. When the former has escaped out of the canula externally, the latter is to be drawn out entirely, (see Fig. 10.) The sound with the double conduit is now to be introduced, and the process carried on, (as represented in Fig. 11.) as long as the patient can support the presence of the apparatus. In this last part of the operation, it would, without doubt, be preferable to remove the canula, which, from its size and form, would soon be found too irritating to the patient, and to leave only the bag and double sound in the urethra. The double sound should also have a slight curvature, and be as flexible as possible, for obvious reasons. At the close of each operation, the bag should be washed out with simple water previous to the withdrawal of the instruments. Perhaps the only practicable way of withdrawing the bag, after the first operations, if the stone remained of any size, would be to rupture its closed extremity; and in the subsequent operations fresh ones could be employed.

But the great problem yet to be solved, is—of what material are the bags to be made? and how prepared? so that the four great requisites may be attained of strength, thinness, pliability, and ability to resist the chemical action of the various solvents, necessary in different cases? To determine these matters, M. Robinet has already made a great number of trials. He had prepared, under his direction, single, double, triple, and quadruple membranes, and in close juxtaposition, so as to have bags of various strength. His first experiments had a reference simply to the physical resistance of the membranes, and he arrived at the conclusion, that a double one had all the strength requisite. In the next place, he occupied himself in observing the action of various menstrua upon the unvarnished membranes, and introduced into single, double, and triple bags the following liquids;—

1st. A solution of caustic potash, in the proportion of sixteen parts of water to one of alkali.

2nd. Hydrate of lime diffused in water, (lait de chaux.)

3d. Sirupy phosphoric acid, diluted with twelve times its quantity of water.

4th. Common vinegar.

5th. Muriatic acid, diluted with six times its weight of water.

Bags filled with these several liquids were suspended in the open air. At the expiration of twenty-four hours none of them were found to be torn; but they had suffered part of the liquid to ooze through. The single bags contained but a small quantity, the double ones a considerable portion, and the triple ones had scarcely lost any. After some days exposure, in this way, the most of them were rent; but this occurred precisely in that part of the bag, immediately above the liquid, and on which, of course, the corroding liquor acted in a more concentrated degree, by the evaporation of its water. It results, from these trials, that the membranes, of themselves, offer considerable resistance to the action of those solvents, since it was only by simple sweating through, that they escaped during the first twenty-four hours. It may easily be conceived, however, that a bag thus filled, and surrounded by the urine in the bladder, or by any other bland fluid, artificially introduced there, would be placed under much more favorable circumstances, and so far from the caustic liquor oozing into the bladder, and mixing with the urine, we might expect the opposite effect to take place, viz. the urine, by the pressure of the bladder upon it, to ooze into the bag, where there can be no pressure. In this way too, the urine occupying the pores of the membrane, would prevent this last from being so effectually corroded by the caustic liquor.

M. Robinet's next great object was to determine what kind of varnish would render the membranous bags most impervious, and for this end, he likewise instituted many experiments. The character of the solvent must, of course, determine the nature of the varnish. It appeared to him that, that usually employed in varnishing taffeta silk answers the purpose very well for acid menstrua. Accordingly, after having given some of the bags a coat of linseed oil, rendered drying by litharge, he suffered them to dry properly, and submitted them to several trials. Even tolerably concentrated acids had no action whatever upon them; but a solution of potash, in a very short time, took off the coat of varnish.

Gum Caoutchouc likewise attracted the attention of M. Robinet; but with a full view of all the difficulties attending its employment. Its application was nevertheless attempted by several processes. He successively dissolved the gum elastic in sulphuric ether, in the volatile oil of turpentine, in linseed oil, and lastly in a peculiar oil sent to him by M. Payen, which has the property of dissolving it even when cold. By means of these different preparations, he succeeded in rendering the bags incapable of being acted upon by any of the solvents; but they all had one weighty inconvenience—it was found impossible to dry them so that they would not stick when the surfaces came into contact. M. Robinet, however, with a rare and truly philanthropic zeal to attain the desired object, was not to be deterred—and after diversifying the mixtures of the above solutions, he had recourse to alcohol, which has, as it is known, the property of precipitating caoutchouc from its solution. He hoped, by the agency of this fluid, to dissolve the portions of volatile oil, still in connexion with the varnish, and thereby to leave the caoutchouc pure at the surface of the membrane. But success still eluded his pursuit. He expected after some time to continue his essays, with the juice of the tree which produces the gum elastic; when, it is to be hoped, the proper result may be obtained. We have not had an opportunity of learning, within these few years back, what has been further done on this important subject.

He proposed to himself, as a last subject of inquiry, to ascertain, 1st. What are the best menstrua for the various species of calculi? 2dly. How much time must be employed in dissolving a calculus of a given volume and weight? 3dly. Whether there is any difference, and in what it consists, with regard to the solubility of recent and dried calculi? 4thly. What advantage can result from the employment of the different solvents at different degrees of temperature. 5thly. What benefit might result from the substitution of a current of the dissolving liquor, in the place of allowing the calculus to remain in a certain quantity of it for some time? Difficulties would, of course, arise in the cases of calculi having alternating layers of different substances, requiring different solvents; as well as in ascertaining with positive certainty, the exact chemical character of a calculus, whilst in the bladder. The analysis of the urine, and the general diathesis, might throw much light upon each case in this respect; but it is to be feared they would not afford that precision of information so necessary in such a method of treatment. With regard to the simple application of the instruments to the living bladder, (should every other indispensable requisite be successfully responded to) there would scarcely appear to be more difficulty than in the case of Dr. Civiale's method of lithotrite. In concluding the notice of M. Robinet's plan, might not it, (if eventually found practicable) and the "operation Civiale," assist each other occasionally? In cases, for example, of extremely hard and voluminous calculi, might not the latter, by a first essay, expose enough of their detritus to give information of their chemical character? When the former might be brought in to complete the noble work of destruction; and thus, indeed, accomplish the first conception of Dr. Civiale. The anticipation of many an undoubted, and indeed, familiar fact, of the present day, would have equally been deemed, twenty years ago, "une espece de chimere."

Lest any thing should be left undone in such a cause—lest a *single stone* should be left untouched in the glorious field of investigation, we might say, of destruction, MM. Prevost and Dumas proposed, but a few years ago, to effect the destruction of calculi, by placing them in the circuit between the poles of a galvanic battery. The two conducting wires were to be insulated, in their passage to the inside of the bladder, by being introduced through a gum elastic sound with a double conduit. The agency of such a principle, we all know to be powerful enough for almost any purpose, (certainly for the purpose of disintegrating, as well as breaking up the chemical character of calculi in the laboratory); but to the enlightened practitioner of medicine and surgery, what a host of formidable, insurmountable obstacles array themselves at once against its application in the living human bladder! The names of such men, engaged in even such a hopeless pursuit as this, merit not the less, the meed of praise; but when the most learned and dexterous experimenters are thus driven to extremity, in their laudable and merciful exertions, a correct estimate may be formed of the painful perplexity of the case. The old saying, 'there is nothing new under the sun,' happened to be true of this method likewise—for some ten years before MM. Prevost and Dumas gave their views to the scientific world, Dr. Gruithuisen of Bavaria, had not only proposed a similar mode, but had publicly insisted, that he could succeed with 600 galvanic disks; and Mauduit, of Varenne, had, still previous to that, advised a trial of the agency of electricity.

Previous to our few remarks on the method of lithotrite, it may not be amiss to take a cursory glance at the practice of the Egyptians, of mechanically dilating the urethra, during the time of Prosper Alpin, at the close of the 16th century. He himself was a witness of the practice he describes. He relates the case of a military chieftain, from whom several stones were, in this way, extracted by a distinguished Arab. He made use of a canula of wood, in length about 8 diameters of the finger, and of the thickness, or diameter of the thumb—With this, he blew with force into the canal of the urethra—to prevent the air from penetrating into the bladder, he took care to press strongly, with his other hand, the other extremity of the urethra against the arch of the pubis. After thus forcing air into the urethra, he kept it there by closing the external orifice of the canula—then introducing the finger into the anus, he gradually directed the stone towards the orifice of the neck of the bladder, and from thence into the canal of the urethra. When it was thus brought near the meatus urinarius, he withdrew the canula, with violence, from the urethra, and in this way procured the discharge of a stone the size of the kernel of an olive. Prosper Alpin afterwards assisted at two other operations, practiced in the same manner, by the same man—in one case, that of a child, he withdrew eight calculi; from the other, an adult, he extracted a stone of the volume of a large olive.

He mentions likewise another, and no doubt a better, process, by another Arab practitioner, and which consisted in introducing, successively, into the urethra, a series of flexible and dilatable canulæ, and dilating them by forcing the air into them. We suppose that all, except the last canula, had their inner extremity closed to prevent the entrance of the air into the bladder. The canulæ were of three or four different diameters, and when the last and largest was introduced, the operator passed his finger into the rectum, and endeavored to bring the calculus into the inner opening of the canula; its further entrance into the canula was to be promoted by suction at the external opening. In one case of this kind the stone was broken.

Now we may remark of these processes, that they were sufficiently rude, but if the cases be true, as stated, it might lead the mind to inquire into what extent the urethra might be dilated by more skilful means, were they *slowly* and judiciously employed, for the evacuation of moderately sized calculi, and for preparing the urethra for the introduction of the straight sound in the 'procede Civiale.'

The last method of answering the first great indication, that we will notice, is the "procede Civiale." Within the few years back so much has been said, and written, and done, on this interesting development of modern surgery—it is now so completely the property of the surgical world, that but little need be said of it, and that in very general terms, in addressing ourselves to the learning and intelligence of the profession.

Dr. Civiale, between the years 1817 and 1824, (the period of the first conception and ultimate perfection of his processes) was one, (of the many distinguished men in the records of history) who, with talents and devoted zeal, had to contend against the insufficiency of fortune, and the prejudices always springing up in opposition to every thing of a novel character. In the year 1818 he called upon the French government for aid in prosecuting his inquiries, and his memorial was referred to the Royal Academy of Sciences, as the body most capable of determining the merits of the case. But the committee appointed by that Academy, refused to report at that time, and of course aid was denied. The same committee reported favorably in 1824, when the question had been settled by extraordinary individual enterprise, and when, of course, aid was no longer wanting. We mention these facts as illustrative of the honorable disposition of the French government to patronise science, as well as to show the occasional strange fatuity that sometimes comes upon even learned societies, with regard to the dearest *interests* of the sacred cause in which they are engaged. Arriving at the fruition of all his anxious expectations, Civiale now consoled himself by the thought of having done well for his species, and by indulging, in delightful anticipation, the award of posterity. But in examining closely the chaplet of his fame, he discovered the loveliest flower missing, the merit of originality. Gruithuisen, whom we have mentioned before, had, in the year 1813, published a rude and undigested, *but similar* plan. It was, however, merely proposed, and almost forgotten, and but for the genius and enthusiasm of Civiale, might have been forever swallowed up in the fathomless gulph of the past eternity. To claim the honor of discovery for Gruithuisen, would be about on a par with awarding to the Spaniard, Blasco de Garay, the reputation of determining, at Barcelona, 290 years ago, the practicability of propelling vessels by steam; or in still later times, with less justice, to Savery, and to Hulls: shades of our renowned countrymen! spirits of Evans and of Fulton! what had been the present condition of this Western habitation of civilization and learning, had not you become the virtual, the practical discoverers of steam navigation; at the commencement, even at the risk of appearing as fools for science's sake. We will not advert to individual opposition to the 'procede Civiale,' although that was not sparingly dealt out in France and elsewhere.

Century after century had rolled on, from the first dawn of surgical knowledge, in pursuit of a safe method of destroying stone whilst in the bladder. The various modes had their day of vogue, and were successively laid aside, and only recorded in the annals of medicine, by way of caution, and of sparing useless labor to future investigators. To hope for success, by the application of any principle within the present scope of man's knowledge, seemed almost to be hoping without hope. It was reserved for our own time to solve the great problem, and to show that it can, at least in many cases, be effected, and that too, according to the simplest principles of mechanical philosophy.

It is not necessary, as has been already remarked, to enter here into a description of the instruments, or of the mode of employing them. Suffice it to say, that their ingenuity and practicability have been extensively tested by experiment, and that, *so far*, the 'operation Civiale' is the only one that does answer effectually, in many, perhaps most cases, the first merciful indication. We do not know the number of cases that have occurred in Dr. Civiale's practice of lithotrite, since the close of the year 1826, when he published his work on the subject:—no doubt it has become very considerable. But upon referring to that work, there is abundant evidence to show that the knife should never be resorted to in any case, unless it be first ascertained that that method (or some other equally, or more practicable one *yet* to be discovered) cannot be available.

At the period just referred to, Civiale had operated upon 43 calculous patients, but one of whom died *during* the treatment necessary. Their united ages amounted to 2184 years, which shows an average of within a fraction of 51 years. The case that terminated fatally, we give in the words of Dr. Civiale:—M. Cornu, aged 72—disease of five years standing —treated during 3 months—2 voluminous and very hard calculi—bladder diseased—constitution very feeble—before the end of the treatment, M. Cornu, as a consequence of a deviation from the prescribed regimen, experienced all the symptoms of acute gastritis, which proved fatal. This was demonstrated by a post mortem examination. It was likewise discovered that the bladder *still contained a fragment* of stone, one inch and a half in its greatest diameter, and that this viscus was in the state in which it is ordinarily found, when the disease has been of long standing. With regard to the volume and texture of the calculi in all the other cases, there appeared to be such a variety as might have been expected, in such a number of patients. They varied from the size of a hen's egg, down. The larger size and greater hardness constituted, of course, greater difficulties, and required more frequent operations.

*Perhaps it is fairer to test the advantages* of this method by the first few years' practice, than by that of subsequent periods, as it was then novel, and no patients, but those whose chances of success by other methods were diminished, by the ravages of age, or by great constitutional debility, induced by their long standing calculous affections, or from any other cause, could be brought to submit to it.

It would be expecting too much of the method of Civiale, as of any other process that could possibly be devised, to suppose that it would be exempt from difficulty. There must of necessity be great difficulty, if not absolute impossibility, to grasp an extraordinarily large stone; and the perplexity of the case would be inevitably increased were it very hard.

Fragments too, as in the case of M. Cornu, sometimes remaining, and irritating the bladder, by their sharp angular projections, must be put down as an important point of consideration. If the stone be encysted wholly, or partially, this method is out of the question. Independently of the stone itself, great difficulties, must likewise occur, where there exists considerable morbid irritability of the bladder, and especially where such irritability pervades the canal of the urethra, or where there is a stricture in that canal.

The latter obstacles, however, may no doubt, be generally overcome by cautious and prudent management. With regard to the last, stricture of the urethra, we cannot but direct the attention of the junior members of the profession, to the wise, and prudent, and *slow*, and successful practice of the chief surgeon of the Hotel-Dieu of Paris, Professor Dupuytren, in such cases. He always reminded us of Old Fabius, who conquered by delay, whilst younger medical Varros sometimes ruin their cause by their haste and impetuosity. With regard to simple organic stricture, the usual mechanical method of bougies, of increasing diameters, successively worn in the urethra, for 15, or 20, or 30 days, commonly overcame them. Occasionally a bougie could not be made to enter the stricture; but upon being left in the urethra, the end of it in the neighborhood of, or touching the strictured part, it seemed, after some hours of *patient waiting*, to determine an expansive action in the tissue of the part, and *then* the bougie could be insinuated, and left there, to prepare the way for the next larger one. M. Dupuytren always employed, in these cases, bougies of gum elastic, of a conical form, decreasing to a very small extremity. The writer of these observations was frequently interested, in observing in cases of spasmodic stricture especially, the happy effects of the above prudent practice. Sometimes when the smallest bougie could not be made to enter the obstacle, the presence of even a large gum elastic sound for seven or eight hours, as before explained, would prepare the way for the subsequent introduction of the bougie.

Our object, in making the preceding remarks, is to explain how morbid irritability, and even stricture of the urethra, may perhaps in most cases, be *slowly* overcome, so as not only to admit of the introduction of the straight sound, but to bear its presence during the operation. If we take this in connexion with the Egyptian practice according to Prosper Alpin, already adverted to, we know not to what extent the urethra might be dilated by *slow and cautious* means, for the introduction of straight sounds, of even larger diameters than those that have been hitherto employed. We would, in this way, account in some respect, for the failure of many attempts, that have been made in this country, to practice by the method of Civiale, in so much indeed, as to impair the confidence of the American profession, as to its extensive usefulness. It has, no doubt, sometimes happened, that after *hurried*, ineffectual efforts have been made according to that process, recourse has been eventually had to cutting, when judicious management might have insured success. The patients might have been in agony at the introduction and presence of the instruments in the parts, and might have subsequently yielded themselves to harsher treatment; but as these cases seldom call for prompt and rapid action, it might yet remain a question, whether nature might not have suffered herself to be *coaxed* by degrees, in her own way, into subserviency to our purposes.

Various modifications of Civiale's instruments have been proposed and even practiced with by others; but it does not come into our plan to enter into an account of them, particularly as (so far as they have come to our knowledge) they have been found to be less ingenious and less practicable than those of Dr. Civiale.

We will now briefly consider some only of the means that have been practiced to meet the second great indication, viz. cutting a passage for the exit of calculi. It was a part of our plan, to take a rapid glance at the history of *Lithotomy*, in order to bring into comparison the various methods that have at different epochs been devised and adopted;—to trace some of the more remarkable improvements that have from time to time been added to the resources of surgery, and especially the improvements of later times;—to distinguish what in fact is new from what is old;—to collect together (as far as time, and the limited means within our reach of consulting the annals of medical history would allow) the relative average of successful cases in each mode of practice;—in a word, after a careful review of all, to sum up in favor of some method, or methods in particular, which might afford the best chances of safety to the patients themselves, and reflect most credit upon the profession. Time however, and the limits we prescribed to ourselves, would not admit of it.

As no operation has more exercised the genius of medical men in every age than that of lithotomy;—as the walls of the bladder have been attacked by instruments on all accessible points;—as the great embarrassment consists in the proper appreciation of the divers methods, so as to form a correct estimate of the circumstances in each case, that should determine in favor of one method in preference to another, or, if possible to ascertain whether *one mode* in particular, might not be found to combine within itself all the requisites for the generality, if not for all the cases, that *require* the aid of lithotomy;—such a research, comprising a review of all the questions bearing upon this department of our subject, constituting of itself a sufficiently extensive field of investigation, would, in the hands of some more skilful laborer in the vineyard of medical science, no doubt, be highly acceptable to the profession, and prove eminently useful to the sacred cause in which we are all engaged. We will confine our few observations to those operations that have been practiced in more modern times.

The high operation, or that performed above the pubis, was probably the mode employed by Colot, in the case of a criminal laboring under stone, as far back as the year 1475. It is reported, that the criminal recovered in 15 days, and was pardoned by Louis XI. But the first well authenticated operation was one practiced by Pierre Franco, at Lausanne, in 1560, on a child 2 years of age. It was a case of necessity. He commenced by the operation of Celsus; but the stone being as large as a hen's egg, and seeing the bladder cause a considerable prominence above the pubis, he made the incision there. The case was successful, but he still advised others not to follow up such practice, on account of the danger, now always apprehended—*urinary infiltration.*

In after times the improved processes of Cheselden and Frere Come seemed, at least in their hands, to be sufficiently successful: for of 15 cases, in 4 years time, occurring in the practice of Cheselden and Douglas, but 2 patients perished; and out of 82 operations by Frere Come, almost all the patients were cured. In more modern times, however, the proportion of successful cases to those not so, appears to be only four to one. This operation does not present the danger of cutting any important vessels:—in upwards of 80 cases, Frere Come encountered but one single hæmorrhage. But if this advantage be taken into consideration, on the other hand, the patient is exposed to all the evil effects of urinary infiltration in the cellular tissue, and consequently of inflammation of the peritoneum, &c., accompanying it. At this time, the high operation is confined to those cases, where the extraction of voluminous calculi is thought to be impossible, by the methods employed below the pubis.

The common lateral method, we know, was first practiced by Frere Jacques, in a very rude manner, towards the close of the 17th century. Out of 42 cases at the Hotel-Dieu and 18 at the Charity hospital, making 60 in all, 25 patients died. In several of these cases he had cut into the rectum. This method, however, has successively undergone many essential improvements, in the hands of Rau, Cheselden, Bromfield, Frere Come, Hawkins, and others, and at this time, we believe, the number of deaths in Europe is about one in six: in America, however, from some cause or other, the operation seems to be less formidable in its character, as the mortality is evidently in considerably less proportion. The lateral method exposes less to inflammation than the high operation; but there is much greater danger of hæmorrhage. If the surgeon approaches too near the bulb of the urethra, he may probably wound the arteria transversalis perinæi. Should the incision incline too transversely, he may even run the the risk of dividing the internal pudic artery. As for the superficial artery of the perinæum, frequently no precaution can save it. To this may be added, the danger of cutting into the rectum, when the incision is carried too far backwards. Young practitioners should be very careful of this latter circumstance, and should operate several times upon the dead subject, before trying their skill on the living. We remember well the *sad* accidents of this sort, that befel many of the *dead subjects*, in the practice of some of the members of the class of operative surgery, whilst we attended it under Professor Lisfranc, at the Pity hospital of Paris. In the lateral method the lithotome cache of Frere Come is now generally employed in France, and we believe, frequently in England. The easy and safe mode of introducing the lithotome, and then of directing the incision from within outwards; the perfect control which even an inexperienced hand may have over the instrument, certainly gives it decided advantage over the gorget, and should recommend it likewise to the American Profession. With regard to the two preceding methods, and the improvements that have been successively added to them, down to our own times, they are matters of sufficiently general medical notoriety, not to insist farther. Our object has been simply to lay down at first the advantages and disadvantages (according to the general consent of scientific practitioners) of methods still in use, but long known, and then rapidly to contrast them with the real, or supposed claims of methods peculiarly of our own day, and not so generally known.

A new mode of operating was proposed in the year 1818, and has since been practised with various success by M. Sanson, called the Recto-vesical method. But we are told at the outset, that we should call things by their right names, and style it the vertical method, or the grand appareil, or the method of Marianus Sanctus, of the sixteenth century, revived. That the external incision should be made along the median line of the perinæum in both the old and new methods, is only one point of resemblance, and cannot subject the author of the new one to assuming credit (whatever may be the credit eventually awarded to it by the profession) to himself for the labors of antiquity. Besides Marianus Sanctus did not in truth make his incision on the median line; but a little to the left of the raphe. The subsequent stage of the old operation, was simply to dilate at once, the neck of the bladder and the neighboring part of the urethra, to the degree necessary for the exit of the stone, and this, of course, could only be effected by the *laceration* of the parts. We need scarcely advert to the introduction of a grooved staff, or catheter to serve for the direction of the other instruments, as another point of similarity, as this is common to all the methods below the pubis of the present day. Surely no enlightened practitioner of the 19th century would wish, even if he could do so without detection, to encircle his brows, with the honor of having invented so cruel an operation. It suited the period of its vogue; because surgery could then offer nothing better.

The recto-vesical method of M. Sanson is simply this, a grooved catheter being introduced into the urethra, the operator makes a longitudinal incision along the raphe, commencing at, or near the anus, and extending forwards in length, of course, according to circumstances, and divides the membranous portion of the urethra: then introducing a probe-pointed bistoury into this opening, and directing the end of it along the groove, he divides the prostate gland, and the neck of the bladder and a portion of its inferior wall. The stone is extracted as usual. Sometimes the anterior and inferior portion of the rectum and the sphincter ani are involved in the incision, which, beyond all doubt, should be avoided, if possible. M. Dupuytren has several times practised this operation; but he employs the lithotome, making the incision of the neck of the bladder, &c., in withdrawing the instrument, which would we think always be preferable.

Professor Vacca Berlinghieri has slightly modified this process, and after the end of the bistoury has been made to enter to the distance of about one inch into the interior of the bladder; without changing the position of the catheter, he raises the handle of the bistoury to a distance, required by the necessity of the case, in the direction of the scrotum. By this motion, the back of the blade resting upon the groove of the staff, the end of the bistoury, within the bladder, must separate from the groove, and the cutting edge of the blade must necessarily divide the neck, prostate gland, &c. In this position of things, the bistoury is withdrawn. The recto-vesical method has been frequently practised in Italy, and, it has been reported, with considerable success. In France, experience had been too limited, when we left that country, to warrant an ultimate decision as to its merits. It will strike every medical reader, that the incision directed along the median line, goes out of the reach of any of the arterial trunks, and that, of course, there can be no fear of hæmorrhage. Practice has confirmed this advantage. Neither is danger from inflammation to be apprehended. These are weighty circumstances, that preponderate much in its favor over either the hypogastric, or lateral method. But its friends are not blind to its great and perhaps only radical defect, its exceeding liability to occasion urinary fistulæ; which cannot but be viewed as one of the most incommoding, nay distressing and, in many cases, permanent evils that could be consequent upon lithotomy.

We will conclude our observations by noticing the transverse, or bi-lateral method, as practised by Professor Dupuytren at the Hotel Dieu, of Paris. Again must we name this the method of Celsus, rescued although it may have been indeed from the wreck of ages; because, forsooth, the incisions are transverse. But the Roman simply directed the surgeon to go forth to the aid of suffering humanity, armed with a bistoury in his right hand, and the fingers of his left. At the close of the operation, to be sure, if his fingers were inadequate to the extraction of the stone, a crotchet, of a peculiar construction, was to be brought to his aid. The Augustan age had, however, been stripped of its laurels by MM. Chausier and Ribes, already in the 19th century, (1805) and Beclard had practised upon the living and the dead as late as 1813. It had been, at the first period, forseen that a conductor was only wanting to direct the incisions with safety, that the Celsian mode might become a good one even in modern times.

After the first incision (the catheter being previously introduced) the urethra was to be cut into as usual—then the neck of the bladder and prostate gland were to be divided, on one side *first*, and *then* on the other—or indeed both might be divided at once with a double lithotome cache, like that of Fleurant,* more anciently described by Pierre Franco. As far as our limited research could extend, we have not been able to ascertain the fact, that Pierre Franco ever did invent a double lithotome cache. It is certain that he did, however, propose a single one, to all intents and purposes similar to that of Frere Come. We must then cease calling this last instrument by its accustomed name, and style it the lithotome cach of Pierre Franco, because, although it had slept in quiet oblivion for two centuries, it still owed its discovery to him. With about the same reason would we fasten upon the distinguished individual who has added to the materia chirurgica the beautiful instrument which now goes under his name, the surgical plagiarism of having taken credit to himself for the crude and undigested labors of long past, as well as of recent times. Whatever may be the merits of the question, these late developments seemed, even in the short period up to 1824, to have been already withering under the wasting influence of premature old age. Even the keen and searching eye of a Dupuytren had not been able to penetrate through the thick shades that had already begun to invest them with their oblivious mantle, even in the city of their birth. The vigorous intellect of that man of the age, it appears was wanting to act upon the same subject—his ingenuity, and skill, and dexterity, were to be exerted to give it celebrity, to convince men of its practicability, its safety, its usefulness; or the world might have rolled on for two centuries more, before some fortunate *discoverer* might have illustrated his name with the labors of the beginning of the 19th century.

The instruments required in the method of Dupuytren are, an ordinary grooved catheter, a bistoury, and a double lithotome cache. The form of this last nearly resembles that of

* Richerand Histoire des Progrès Récens de la Chirurgie.

Frere Come: it has, however, two blades, curved on their sides, and which, lying upon each other, and concealed in the body of the lithotome, are prevented from wounding the canal of the urethra, &c., when the instrument is introduced. These are made to separate from the body of the instrument by pressing two levers, that extend backwards on each side of the handle, to the handle itself. The handle is of a conical form, and is moveable on a graduated screw, and so disposed, that the separation of the two blades can be regulated at will. The cutting edges of the two blades, during the separation, describe also a slightly curved direction, so that the whole incision will have a curvature, whose convexity will look upwards and forward. In the procede Dupuytren, as in every other, the operator must be provided with director, forceps, &c.

The manner of conducting the operation is this: the patient being placed and secured in the position required for the lateral operation, a grooved catheter is introduced, as in the ordinary way also, and kept in a vertical position by the assistance of an aid. With his bistoury the operator makes, across the raphe, about half an inch above the anus, a transverse incision, of about half an inch in length, with a slight curvature, whose concavity looks backward, and divides the skin and cellular tissue. A second incision divides the inferior wall of the urethra. The bistoury is then laid aside, and the extremity of the lithotome is made to pass through the opening of the urethra into the groove of the catheter, and along the course of this into the bladder. The catheter being now withdrawn, the lithotome is held in such a manner that the concavity of its curvature looks downwards and backwards, which was the very reverse in passing the instrument into the bladder. The two levers are then pressed against the handle, which has been previously turned to the requisite degree on the screw, and the whole instrument is withdrawn, cutting on each side of the neck of the bladder, the two lateral lobes of the prostate, and a small part of the membranous portion of the urethra, which is cut after the fashion of a pen. The stone is then extracted as usual.

Reasoning upon the prima facie evidence of the operation, by way of summing up its probable advantages, in comparison with those of the other modes already remarked upon, it would appear to possess a much greater tendency to spare the rectum, than either the recto-vesical, or lateral method. In fact, it cannot easily be conceived how the rectum can be reached in any case, unless it should arise from absolute carelessness on the part of the operator. The convex side of the blades being turned upwards and anteriorly, the incision must necessarily be removed from the rectum. The whole incision being divided between the two sides of the median line, its extremities are but slightly removed from it; hence the arterial trunks might be considered in less danger than in the lateral method. The danger of consecutive inflammation cannot be greater than in either of the two above-named modes. It would appear to follow, from the division of the incision on each side of the median line, that the operator could give it sufficient extent to afford a passage for the largest calculus, without running much risk of reaching important arteries; and hence, the necessity of ever recurring to the operation above the pubis might be obviated. The manner in which the incision is made from within outwards, would, to our view at least, seem preferable to the mode of conducting the common lateral operation with the gorget, or bistoury, as well as making the incision with a bistoury in the recto-vesical. Experience, however, the great arbiter of all human inventions, must also determine in this.

Since the close of the year 1827, we have not ascertained what has been the voice of that experience. Previous to that time, however, whilst we were attending the clinical instruction at the Hotel-Dieu, of Paris, a rule had been established in that hospital, of submitting the calculous patients alternately to the three mothods below the pubis, with the view to test their relative superiority. There could, in this way, be no choice of patients for any one. Before we left the country, the whole number operated upon under that rule was 21, among whom there was such a variety of age and circumstances of constitution, as might have been expected. It had been agreed that M. Dupuytren should employ the transverse; M. Sanson, second surgeon of the hospital, the recto-vesical; and M. Breschet, surgeon in ordinary to the same establishment, the common lateral method. Out of the 7 cases operated upon according to each process, the transverse was attended with entire success; 2 patients perished that had been submitted to the recto-vesical, one of whom (65 years of age) had been, a month previously, subjected to the process of lithotrite by M. Meirieu, all the other circumstances of his case were, to the last degree, unfavourable; M. Breschet lost one patient. Previous to the commencement of this trial, M. Dupuytren had performed the transverse operation two or three times with complete success.

## Figures and Tables

**Figure f1:**
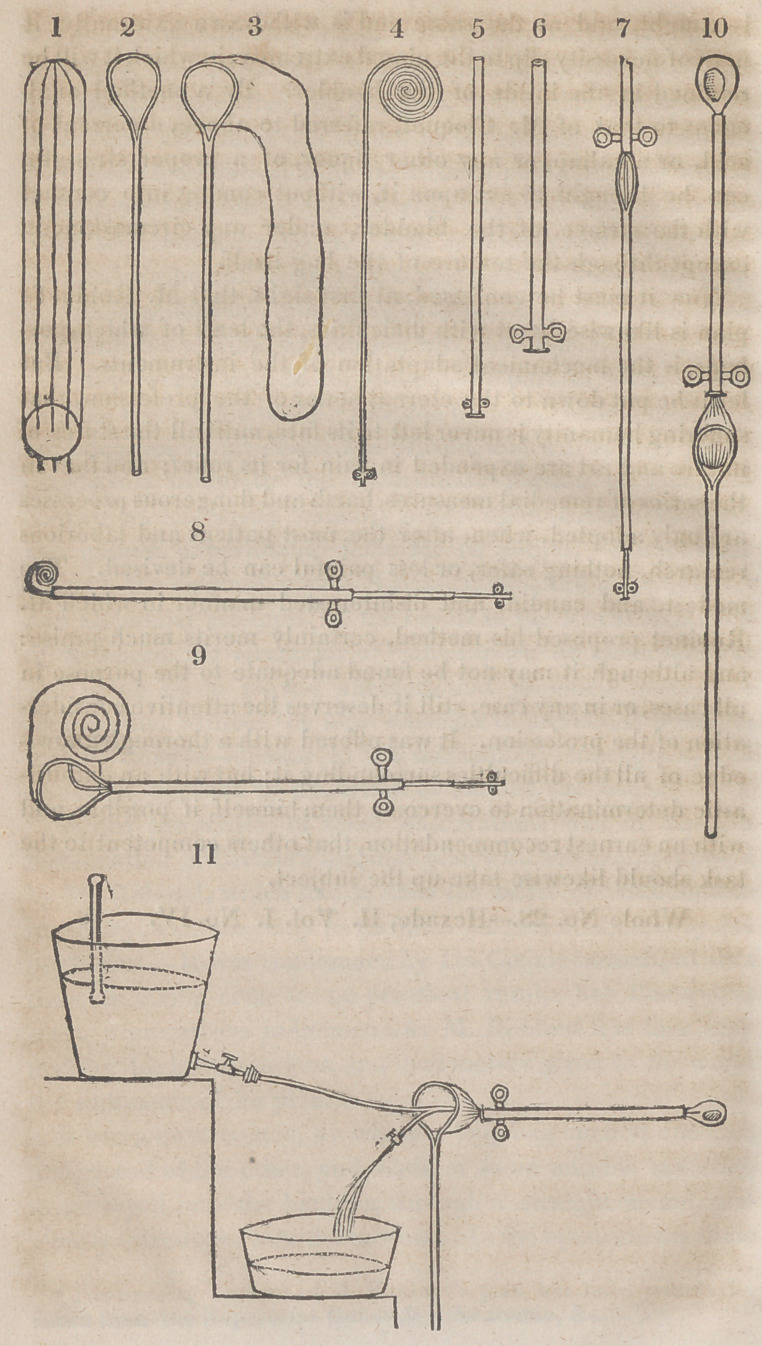


**Figure f2:**